# Effect of a school-based oral health education in preventing untreated dental caries and increasing knowledge, attitude, and practices among adolescents in Bangladesh

**DOI:** 10.1186/s12903-016-0202-3

**Published:** 2016-03-25

**Authors:** Syed Emdadul Haque, Mosiur Rahman, Kawashima Itsuko, Mahmuda Mutahara, Sakisaka Kayako, Atsuro Tsutsumi, Md. Jahirul Islam, Md. Golam Mostofa

**Affiliations:** UChicago Research Bangladesh, Dhaka, Bangladesh; Global Health Promotion, Division of Public Health, Graduate School of Medicine Tokyo Medical and Dental University Yushima, 1-5-45 Bunkyo, Tokyo, 113-8519 Japan; Honjo International Scholarship Foundation, 1-14-9 Tomigaya, Tokyo, 151-0063 Japan; Social Science Group, Wageningen University, 6708 PB Wageningen, The Netherlands; Faculty of Policy Studies, Chuo University, 742-1 Higashi-Nakano, Hachioji-shi Tokyo, 192-0393 Japan; United Nations University-International Institute for Global Health (UNU-IIGH), UKM Medical Centre, 56000 Cheras, Kuala Lumpur, Malaysia; School of Criminology and Criminal Justice Mt Gravatt Campus, Griffith University, 176 Messines Ridge Road, Mount Gravatt, QLD 4122 Queensland Australia; Department of Population Science and Human Resource Development, University of Rajshahi, Rajshahi, 6205 Bangladesh; Global Health Promotion, Division of Public Health, Graduate School of Medicine, Tokyo Medical and Dental University, Yushima 1-5-45, Bunkyo, Tokyo, 113-8519 Japan

**Keywords:** Dental caries, School-based health education, Adolescents, Bangladesh

## Abstract

**Background:**

There is a dearth of published literature that demonstrates the impact and effectiveness of school-based oral health education (OHE) program in Bangladesh and it is one of the most neglected activities in the field of public health. Keeping this in mind, the objectives of this study were to assess the effectiveness of OHE program in: 1) increasing oral health knowledge, attitude, and practices and 2) decreasing the prevalence of untreated dental caries among 6–8 grade school students in Bangladesh.

**Methods:**

This intervention study was conducted in Araihazar Thana, Narayanganj district, Bangladesh during April 2012 to March 2013. The total participants were 944 students from three local schools. At baseline, students were assessed for oral health knowledge, attitude and practices using a self-administered structured questionnaire and untreated dental caries was assessed using clinical examination. Follow up study was done after 6 months from baseline. McNemar’s chi-square analysis was used to evaluate the impact of OHE program on four recurrent themes of oral health between the baseline and follow-up. Multiple logistic regression analyses were used to determine the impact of the intervention group on our outcome variables.

**Results:**

Significant improvement was observed regarding school aged adolescents’ self-reported higher knowledge, attitude and practices scores (*p* < 0.001) at follow-up compared with baseline. The prevalence of untreated dental caries of the study population after the OHE program was significantly (*p* < 0.01) reduced to 42.5 %. Multiple logistic regression analyses showed that the OHE intervention remained a significant predictor in reducing the risk of untreated dental caries (adjusted odds ratio [AOR] =0.51; 95 % confidence interval [CI] = 0.37, 0.81). In the follow-up period participants were 2.21 times (95 % CI = 1.87, 3.45) more likely to have higher level of knowledge regarding oral health compared to baseline. Compared with baseline participants in the follow-up were 1.89 times (95 % CI = 1.44–2.87) more likely to have higher attitude towards oral health. In addition, OHE intervention was found to be significantly associated with higher level of practices toward oral health (AOR = 1.64; 95 % CI = 1.12, 3.38).

**Conclusions:**

This study indicated that OHE intervention was effective in increasing i) knowledge, ii) attitude, and iii) practices towards oral health; it also significantly reduced the prevalence of untreated dental caries among school aged adolescents from grade 6–8 in a deprived rural area of Bangladesh.

## Background

Oral health is a core component of general health and well-being. A healthy mouth enables an individual to speak, eat, and socialize without experiencing active disease, discomfort or embarrassment [1, 2]. Few aspects of health are as accessible to personal control as oral hygiene, which can be improved by simple behavioral changes. Oral Health Education (OHE) program, which has as its objective, the improvement of the oral hygiene status of the participants would have obvious merits. Improvement in knowledge as a result of OHE has been known to influence not only self-reported oral health related practices and behavior in a favorable way, but also improve clinical parameters of oral health such as oral hygiene, gingival health and dental caries [2].

Oral health education and promotion may be delivered at multiple forums namely, hospitals, primary health care centers, private dental clinics as well as school. Yet, schools are perhaps the best place for promoting oral health because approximately one billion children worldwide spend most of their daytime life there [2]. Schools provide an ideal setting to deliver OHE in combination with preventive services to achieve oral health promotion. School based approach has been reported to be more efficient in delivering preventive and curative services than community based approach [3]. Perhaps, school aged adolescents are in particular need of preventive program to ensure positive long-term dental health and hygiene. However, due to lack of health education and insufficient preventive measures, there is high prevalence of morbidity and the health status of these students is not good always [4]. Study found that school aged adolescents who are suffering from poor oral health were 12 times more likely to restrict-activity days compared to those are in good oral health [1, 5]. More than 50 million school hours are lost globally because of poor oral health. This can affect student’s class room performance and success later in life [2, 6, 7].

Bangladesh, a developing country, faces many challenges in delivering oral health needs. There is a big gap in oral health related knowledge and behavior among this country’s population especially among the school aged adolescents [2]. A study in Bangladesh, found poor oral hygiene and bleeding gingiva in 44.3 % of 12-year-old students [8]. Another study showed the crude prevalence of gingivitis and plaques were 17.5 % and 56.0 % respectively among 6–13 years old children [9]. The majority of the Bangladeshi population resides in rural areas and 40 % of the families are constituted by children. These children cannot avail dental facilities due to inaccessibility, financial constraints and stagnation of public dental health care services and therefore are most vulnerable to dental diseases. The country has very limited facilities for dental treatment and a high population to dental provider ratio (100,000: 2) [10]. In such a population with poor oral hygiene and limited resources for oral health care, interventions that promote improved oral hygiene in adolescents are therefore urgently needed. Oral health promotion programs in schools can be an ideal setting to deliver oral health education in Bangladesh.

Oral health educational intervention has been successful in many developing [11–14] and developed nations [15, 16] around the world. For example, health education campaign among school students in China ‘Love Teeth Day’ was effective for better oral health [14]. ^“^Love Teeth Day” showed declining caries in provinces where prevention activities was run. Another study conducted among Taiwanese adolescents showed a school-based OHE program improved knowledge and behaviors in junior high school students [15].

There is a dearth of published literature that demonstrates the impact and effectiveness of school-based OHE program in Bangladesh and it is one of the most neglected activities in the field of public health. Until and unless, the impact of a program on the targeted population is not determined, the success of the program cannot be assessed. Keeping this in mind, the present study was undertaken to determine the impact of school based OHE program in (1) preventing the prevalence of untreated dental caries and (2) increasing knowledge, attitude, and practices regarding oral hygiene among 6–8 grade school students in Bangladesh.

## Methods

### Study design and participants

This intervention study was conducted in Araihazar Thana, Narayanganj district in Bangladesh. Araihazar Thana is located 25 km south-east from the capital, Dhaka. The total area of this Thana is 183.35 km^2^ with 63,080 house-hold units and a population of 331,556. Araihazar has 12 Unions/Wards, 182 Mauzas/Mahallas, and 315 villages. Males constitute 51.7 % of the population, and females 48.3 %. Araihazar has an average literacy rate of 53.0 % (7+ years of formal education), compared to the national average of 68.4 % literate [17].

Of 26 high schools (grade 6–10) in the study area, two were full government schools and 24 were semi-government schools. We selected one of the government schools and two of the 24 semi-government schools using a simple random sampling method (we drew numbers). We only selected three schools due to limited time and resources. These schools were well-established, older schools and conveniently located. One was a girls-only school and the other two were co-educational. The socio-economic, cultural, religious and geographical characteristics of those schools were very similar. The schools were more than 2 km apart. A total of 1000 adolescent students from grades 6–8 were attended the three schools. The Headmaster of school was contacted by the principal investigator through written communication explaining the importance of good oral health and emphasizing the need for school based oral health promotional intervention. Permission was sought to allow investigator to carry out oral health promotional intervention program. A similar letter explaining the condition and seeking consent was circulated among parents/guardians.

Participants were selected using the following criteria: (1) they were in grades 6–8, (2) with no history of previous dental intervention either preventive or curative (3) were not critically ill and (4) had good general health. Furthermore, failure to obtain written informed consent from parents and children requiring any emergency dental treatment were excluded from the study. Children requiring emergency dental treatment were referred to the nearest center for appropriate care. 944 adolescents fulfilled the above criteria and were included in the study.

### Data collection procedures

#### Before the study

Before conducting the interview, we took permission to provide school based OHE intervention. After permission, we conducted a pilot survey of the questionnaire and revised it, as suggested, for the final survey. In accordance with the general guidelines required for a full study, we selected 10 % of the sample (*n* = 95) for a pilot test in one of the schools in the study area [18]. The questionnaire was drafted in English and then translated into Bangla, the national language of Bangladesh. Back-translation from Bangla to English was carried out as a validation exercise before and after the pre-test questionnaire was administered. We also modified the questionnaire based on the results of the pre-test exercise to make it easier to understand and to answer. The baseline survey was conducted in April 2012. Trained research assistants (RAs) read the questions out loud and the participants answered. A group of 12–15 students participated in each exercise led by one RA sitting in the same room, and we requested them not to discuss the survey questions with their peers. After each session, we invited another group to participate in the survey. The room was provided by the school.

#### OHE intervention

After completion of the baseline survey, we hired one experienced dentist who graduated from medical college in Bangladesh and trained 3 Research Assistants (RAs) for this study. Before the survey, we gave 4 days of training to the RAs and one teacher (selected from the participating schools). Dentist prepared OHE materials following the standard guideline by World Health Organization (WHO) [2] and trained the RAs. The training was delivered using a field manual using *Bangla* language. Students received OHE in the same classroom where they were regularly taught in the school. Each interactive 1 h session utilized audio-visual aids (slide projector, dent form model, charts, photo albums, posters, and plaster models) and focused on: structure and functions of teeth; types of dentitions and their significance; number and types of teeth present in each dentition; dietary components and their effects on oral tissues; importance of a balanced diet; etiology, clinical manifestations, treatment modalities; prevention of dental caries, periodontal disease, oral cancer and malocclusion; fluorides; injurious oral habits; effects of orofacial trauma; influence of oral health on general health; importance of brushing teeth twice daily and mouth rinsing; proper tooth-brushing technique (modified bass technique was demonstrated on a dent form model and reinforced at each subsequent visit); and importance of a regular dental visit once every 7 days.

Furthermore, 12 focus group discussions (FGDs) were conducted in the schools so that RAs and adolescents could become well acquainted with each other. In addition, FGDs were conducted in order to evaluate the effectiveness of the intervention using a qualitative approach.

#### After completion intervention

After 6 months of intervention, follow-up data were collected in the schools using the same questionnaire and with the same clinical oral examination of the study participants as at baseline. RAs visited the homes of any students who were not available at school during the follow-up data collection. In students’ homes, RAs provided the questionnaire to the students and spoke with them in a private room in keeping with the data collection method in the schools.

### Ethical considerations

This study protocol was reviewed and approved by the ethical committee of Bangladesh Medical Research Council (BMRC) in Bangladesh. Prior to baseline survey, participants were informed about the study, invited to participate, and informed of their right to decline. Written informed consent was obtained from the parents and verbal consent was obtained from the Head teacher, class teacher, and participants. In addition, we obtained written permission from local Education Officer under the Ministry of Education in Bangladesh for this study.

### Measures

#### Intervention components

##### Untreated dental caries

WHO was defined life dental caries as the sum of the number of decayed, missing, and filled permanent teeth [19]. The presumption that restorations or extractions of teeth are consequences of untreated dental caries only, however, creates problems when applied to adolescents or adult populations [20]. Moreover, it would be difficult for the study participant to recall the reason for having one or more teeth restored or extracted, which could introduces the problem of recall bias. Tooth decay does not necessarily lead to a restoration, nor have all absent or restored teeth been decayed [21]. In the present study, therefore only “decayed” teeth estimated the true untreated caries disease experience, whereas “missing” and “filled” teeth were not considered as untreated dental caries. All participants were examined by a single trained examiner. The students were seated on the chair and examined in day light using mouth mirror and an explorer.

##### Knowledge and beliefs toward oral health

This section of the questionnaire consisted of 10 questions to determine pupils’ knowledge regarding: (1) periodontal disease can affect health, (2) regular tooth brush can protect tooth decay, (3) fizzy soft drinks affect the teeth, (4) use of fluorides prevent tooth decay, (5) gingivitis is a disease that makes your gums bleed, (6) dental caries is not a infectious disease, (7) fruits & vegetables have effects on teeth & gums, (8) sugar causes tooth decay, (9) tooth decay is a disease that destroys your teeth, and (10) healthy teeth means strong and carries free teeth.

The students’ knowledge was scored using a system adopted from previous studies [14, 22]. Each correct response was awarded 1 point, while incorrect or ‘don’t know’ answers received no marks. This gave a total possible score of 10 points. Participants who scored 0–3 points were adjudged to have poor knowledge, those with 4–7 points to have medium knowledge, and those with 8–10 points to have high knowledge. Cronbach’s α was 0.75 for the knowledge instrument.

##### Attitudes towards oral health

Attitude related parts in the questionnaire consisted of 10 questions to determine pupils’ attitude regarding oral health. Each correct response was awarded 1 point, while incorrect or ‘don’t know’ answers received no marks. This gave a total possible score of 10 points. Respondents who scored 0–3 points were adjudged to have poor knowledge, those with 4–7 points to have medium knowledge, and those with 8–10 points to have high knowledge. Cronbach’s α was 0.69 for the attitude section.

##### Practices related to oral health

This section of the questionnaire consisted of nine items assessing student’s oral health practices: (1) frequency of clean teeth, per day, (2) time spent for brushing in minute, (3) cleansing aid used, (4) materials used to clean teeth, (5) frequency of changing tooth brush, (6) type of toothpaste used, (7) mouth rinsing after eating, (8) clean tongue after meal or during brushing, and (9) frequency of eating candy/chocolate/sweets, per day. A score of 2 was given for good oral health practices, a score of 1 was given for fair practices, and a score of 0 was given for poor practices. The maximum score was 18 points. Students who scored 0–5 points, 6–11points and 12+ points were judged to have poor, fair and good practices, respectively. Cronbach’s α was 0.79 for the practice instrument.

We also collected socio-demographic characteristics such as age, sex, parietal education, school types, and socioeconomic index from all participants.

### Statistical analysis

Data were cross-checked for consistency before final data entry using Microsoft Excel. One person entered the data and then cross-checked it with the principal investigator of the study. Descriptive analyses were conducted to determine the socio-demographic characteristics of the respondents. The household wealth index was used as a proxy indicator for household wealth status. The wealth index was constructed from existing data on a household’s ownership of 15 assets and house construction materials as reported by the participants. Each asset was assigned a weight (factor score) generated through principle components analysis, and the resulting asset scores were standardized to a standard normal distribution with a mean of 0 and an SD of 1. Each household was then assigned a score for each asset, and the scores were summed by household. The sample was then divided into population tertiles: poor, middle, and rich.

We used McNemar’s chi-square analyses as the same individuals are measured twice (before and after the survey) to evaluate the impact of an education program on four recurrent themes of oral health between the baseline and follow-up: (1) prevalence of untreated dental caries; (2) knowledge, (3) attitude, and (3) practices toward oral health. The levels of significance were indicated by *P* values. All values of *P* less than 0.05 were considered as statistically significant (two-tailed). Multiple logistic regression analyses were used to determine the impact of the intervention group on our outcome variables for the sample as a whole after adjustment for the baseline levels of risk factors. For fitting the logistic regression models we merged the data sets of the two periods together. Four binary logistic regressions were used. The dependent variable for the first was coded as “1” if the prevalence of untreated dental caries decreased during the follow-up period. The dependent variable for the second was coded as “1” if the prevalence of higher knowledge level increased during the follow-up period. The dependent variable for the third and fourth was coded as “1” if the prevalence of higher attitude and practice level increased during the follow-up period. We entered all covariates simultaneously into the multiple regression models and estimated odds ratios (ORs) to assess the strength of the associations, and we used 95 % confidence intervals (CIs) for the significance testing. We checked multicollinearity in the logistic regression analyses by examining the standard errors of the regression coefficients. A standard error larger than 2.0 indicates numerical problems such as multicollinearity among the independent variables [23]. The standard errors of all of the independent variables in the adjusted model were below 1, indicating an absence of multicollinearity. The analyses were performed using SPSS for Window, release 22.0 (SPSS, Chicago, IL).

## Results

### Descriptive statistics

Table 1 shows the sociodemographic characteristics of the study participants. More than three-fifths of the participants were female (63.2 %) and remaining 36.8 % were male. Approximately 34.0 % of participants were 10–12 years of old and 96.6 % was Muslims. Regarding father’s education, approximately 14.0 % had no formal education, 37.2 % had below primary level of education, 24.5 % completed primary level of education, and the remaining 24.7 % had secondary or higher level of education. With regard to respondent’s mothers’ education, 22.1 % women had secondary and higher level of education. Approximately 35 % adolescents were from government school. From the total sample population, 43.0 % were defined as being rich, 33.8 % middle, and 23.2 % belonged to the low bands of socio-economic index.

**Table 1:** Basic characteristics of the participants (*n* = 944)

Characteristics	n	%
Sex		
Male	347	36.8
Female	597	63.2
Age, years		
10–12	328	34.7
13	300	31.8
14+	316	33.5
Religion		
Muslims	912	96.6
Non-Muslims	32	3.4
Father’s education		
No education	128	13.6
Incomplete primary education	351	37.2
Complete primary education	231	24.5
Secondary and higher education	234	24.7
Mother’s education		
No education	102	10.8
Incomplete primary education	351	37.2
Complete primary education	282	29.9
Secondary and higher education	209	22.1
Types of school		
School I (Government)	329	34.9
School II (Semi-government)	310	32.8
School III (Semi-government)	305	32.3
Socio-economic index^a^		
Low	219	23.2
Medium	319	33.8
High	406	43.0

^a^Constructed from data on household assets, including ownership of durable goods (such as televisions and bicycles) and dwelling characteristics (such as source of drinking water, sanitation facilities and construction materials). Principal components analyses were used to assign individual household socio-economic scores. These weighted values were then summed and rescaled to range from 0 to 1, and each household was assigned to the low, middle or high tertile

Table 2 shows percentage changes in knowledge, attitude, practices, and prevalence of dental caries at follow-up compared with baseline. Regarding participant’s knowledge about oral health, significant differences were observed in almost all the indicators of knowledge variable before and after OHE program except the participants’ knowledge that dental caries is not an infectious disease and that gingivitis is a disease that makes the gums bleeding. Overall, significant improvement (*p* < 0.001) was observed regarding participants’ self-reported high knowledge score at follow-up compared with baseline (75.9 % versus 19.3 %).

**Table 2:** Knowledge, attitude, and practices regarding on oral hygiene and prevalence of untreated dental caries among 6–8 grade school students (*n* = 944)

Measures	Baseline		Follow-up		Percentage change (%)	*P*-Value
	n	%	n	%		
Knowledge regarding oral health						
Periodontal disease can affect health	432	45.8	830	87.9	42.1	<0.001
Regular tooth brush can protect tooth decay	413	43.7	847	89.7	46.0	<0.001
Fizzy soft drinks affect the teeth	310	32.8	740	78.3	45.5	<0.001
Use of fluorides prevent tooth decay	305	32.3	801	84.5	52.2	<0.001
Gingivitis is a disease that makes your gums bleed	408	43.2	470	49.8	6.6	0.059
Dental caries is not a infectious disease	380	40.2	460	48.7	8.5	0.079
Fruits & vegetables have effects on teeth & gums	399	42.2	753	79.8	37.6	0.003
Sugar causes tooth decay	260	27.5	699	74.0	46.5	<0001
Tooth decay is a disease that destroys your teeth	304	32.2	645	68.3	36.1	0.004
Healthy teeth means strong and carries free teeth	367	38.9	875	92.7	53.8	<0.001
Knowledge and beliefs grading						
Poor (0–3)	523	55.5	88	9.3	−46.2	<0.001
Medium (4–7)	237	25.2	140	14.8	−10.4	
High (8–10)	184	19.3	716	75.9	56.6	
Attitudes toward oral health						
It is important to take care of owns teeth	390	41.3	784	83.0	41.7	<0.001
Needs to visit dentists for dental disease	360	38.1	690	73.0	34.9	0.003
Dentist care only treatment not prevention	240	25.4	320	33.9	8.5	0.098
Clean teeth is one’s duty	355	37.6	790	83.7	46.1	<0.001
Immediate replacement of missing natural teeth by artificial teeth is necessary	140	15.0	255	27.0	12.0	0.059
Treatment of toothache is as important as any other organ in body	201	21.3	710	75.2	53.9	<0.001
Consider oral health as priority	190	20.1	749	79.3	59.2	<0.001
Necessary to brush teeth after each meal	187	19.8	680	72.0	52.2	<0.001
Feel a dental wing is necessary	210	22.2	374	39.6	17.4	0.040
Avoiding smoking is necessary to protect teeth	145	15.4	610	64.6	49.2	<0.001
Attitude grading						
Poor (0–3)	601	63.7	230	24.4	−39.3	<0.001
Medium (4–7)	204	21.6	168	17.8	−3.8	
High (8–10)	139	14.7	546	57.8	43.1	
Practices toward oral health						
Frequency of clean teeth, per day						
1 time	590	62.5	149	15.8	−46.7	0.001
2 times	250	26.4	365	38.7	12.3	
3 times or more	104	11.0	430	45.6	34.6	
Practices toward oral health						
Time spent for brushing, minute						
Less than 1	99	10.5	47	5.0	−5.5	0.041
1	303	32.1	149	15.8	−16.3	
2	289	30.6	348	36.9	6.3	
≥ 2	253	26.8	400	42.3	15.5	
Cleansing aid used						
Toothbrush	275	29.2	795	84.3	55.1	<0.001
Finger	430	45.6	80	8.5	−37.1	
Others^a^	239	25.2	69	7.2	−18.0	
Materials used to clean teeth						
Tooth paste	229	24.3	650	68.9	44.6	0.003
Tooth powder	377	39.9	244	25.9	−14.0	
Others^b^	338	35.8	50	5.2	−30.6	
Frequency of changing toothbrush						
Not using tooth brush	669	70.8	148	15.7	−55.1	0.025
Anytime when it damaged	66	7.0	424	44.9	37.9	
Within 3–6 months	120	12.7	188	19.9	7.2	
After 6 months later	89	9.5	184	19.5	10.0	
Type of toothpaste used						
Not using tooth paste	715	75.7	294	31.1	−44.6	0.001
Fluoridated	42	4.5	439	46.5	42.0	
Non- Fluoridated	187	19.8	211	22.4	2.6	
Mouth rinsing after eating						
Regularly	178	18.9	922	97.7	78.8	<0.001
Irregularly	299	31.7	6	0.6	−31.1	
Not at all	467	49.5	16	1.7	−47.8	
Clean tongue after meal or during brushing						
Yes	270	28.6	847	89.7	61.1	<0.001
No	674	71.4	97	10.3	−60.1	
Frequency of eating candy/chocolate/sweets, per day	88	9.3	398	42.2	32.9	0.043
Less than 1 time	63	6.7	172	18.2	11.5	
1 time	182	19.3	160	16.9	−2.4	
2–4 times	254	26.9	129	13.7	−13.2	
4–6 times	254	26.9	46	4.9	−22.0	
More than 6 times	103	10.9	39	4.1	−6.8	
Practice grading						
Poor (0–5)	419	44.4	177	18.7	−25.7	0.003
Fair (6–11)	347	36.8	248	26.3	−10.5	
Good (12+)	178	18.8	425	55.0	36.2	
Prevalence of untreated dental caries^c^	490	51.9	315	33.4	−18.5	0.001

*Note*: ^a^others = branches of tree; ^b^others = coal, leafs of the tress; ^c^decayed, missing, and filled permanent teeth

There was a significant improvement (*p* < 0.001) at follow-up compared to baseline in higher level of attitude score (57.8 % versus 14.7 %). The highest level of improvement was observed for the indicator that the participants considered oral health as priority (59.2 % versus 20.1 %). Regarding practices, participants who received the intervention reported more frequent teeth cleaning (3 times or more per day) compared to baseline (*p* < 0.001). Significant improvement was observed regarding respondents time spent for brushing teeth (2 min or more), use of tooth brush, use of tooth paste, use of fluoridated toothpaste, rinsing mouth after meal, cleaning tongue after meal at follow-up compared to baseline. Regarding dietary behavior and practices significant improvement was also observed for frequency of eating candy/chocolate/sweets (less than 1 time) at follow-up (*p* < 0.05). Overall significant improvement (*p* < 0.001) was observed regarding adolescent’s self-reported good practices scores at follow-up compared to baseline (55.0 % versus 18.8 %).

The prevalence of untreated dental caries of the study population before the OHE program was 67.5 %. After 6 months of intervention, the prevalence was significantly (*p* < 0.01) reduced to 42.5 %.

### Multivariate analysis

Multiple logistic regression analyses predicting the baseline adjusted post intervention levels of dental caries, and attitudes, knowledge, and practices towards oral health are shown in Table 3. The OHE intervention remained a significant predictor in reducing the risk of untreated dental caries (adjusted odds ratio [AOR] =0.51; 95 % confidence interval [CI] = 0.37, 0.81). In the follow-up period participants were 2.21 times (95 % CI = 1.87, 3.45) more likely to have higher level of knowledge regarding oral health compared to baseline. Compared with baseline participants in the follow-up were 1.89 times (95 % CI = 1.44–2.87) more likely to have higher attitude towards oral health. In addition, OHE intervention was found to be significantly associated with higher level of practices toward oral health (AOR = 1.64; 95 % CI = 1.12, 3.38).

**Table 3:** Adjusted odds ratio and 95 % confidence interval predicting the impact of the follow-up levels of untreated dental caries and high knowledge, attitude, and practices score regarding oral hygiene among 6–8 grade school students (*n* = 944)

Measures	Adjusted odds ratio (AOR)	95 % confidence interval (CI)
Untreated dental caries^a^		
Baseline	1.00	--
Follow-up	0.51^*^	(0.37–0.81)
Higher knowledge level^a^		
Baseline	1.00	--
Follow-up	2.21^*^	(1.87–3.45)
Higher attitude level^a^		
Baseline	1.00	--
Follow-up	1.89^**^	(1.44–2.87)
Higher practices level^a^		
Baseline	1.00	--
Follow-up	1.64^***^	(1.12–3.38)

*Note*: ^a^Models were adjusted by age, sex, mothers’ education, father’s education, types of school, and socio-economic status; here *p < 0.001, **p < 0.01, and ***p < 0.05

## Discussion

The results of this school-based, easy-to-organize, inexpensive educational intervention were found to be effective in increasing school aged adolescents’ self-reported higher: i) knowledge; ii) attitude; and iii) practices scores toward oral health. This study found that OHE program had significantly reduced the prevalence of untreated dental caries. To our knowledge, this study is the first attempt to investigate the effectiveness of the OHE program in preventing untreated dental caries and increasing knowledge, attitudes, and practices regarding oral health among school aged adolescents in Bangladesh. Unlike previous intervention targeting infants [24–26] and preschool children [27] whose oral health is predominantly took care of by their parents, this study focuses on school aged adolescents from grade 6–8 who are at a stage of forming their own health habits [28]. Interventions in this age group are both promising and challenging.

The increase in the knowledge and attitude of the respondents through this intervention could be attributed to many factors; the dentist was well prepared and provided sufficient lectures to discuss the topic more efficiently; at the end of the 4 days of OHE, the trainer reviewed messages with RAs and provided further reinforcement; knowledge and attitude questions were constructed in very simple and understandable manner. Moreover, repeated sessions would probably have brought a better impact in increasing the knowledge and attitude among the participants, as has been emphasized elsewhere [29, 30]. Before the intervention, it was noted that only few of the student were actually visited the dentists for oral diseases.

Another important finding is that after OHE, significant improvement was observed in higher healthy practices toward oral health. The change to healthy practice was occurred by giving adequate information, motivation and practice of the measures to the subjects. The OHE emphasizes the importance of tooth brushing with fluoridated tooth paste; increase the frequency of cleaning teeth at least three times after each meal, and frequency of changing the tooth brush. A giant teeth model was used to help the students visualized the proper way of brushing with fluoridated tooth paste. Another factor that have had contributed to the improvement and retention of proper tooth brushing skill with fluoridated tooth paste was the once a day brushing drill in the school. The factors mentioned above we believe significantly improved and caused retention of the tooth brushing with paste of the students. The issue of retention of practice is very important since most interventions only have short term effects. The study also showed an improvement on the skills of the participants increasing the frequency of rinsing mouth after meal, and cleaning tongue regularly during brushing or after meal.

The school-based OHE program significantly decreased the prevalence of untreated dental caries. School based OHE programs conducted in Brazil [11], Madagascar [12], and Indonesia [13] showed similarly encouraging results. Increasing the knowledge, attitude, and practices toward oral health were key aspects for achieving such desired outcomes in this OHE program. There are several factors that played as a major role in reducing the untreated dental caries in our study. For example, after the OHE being provided, a significant number of students were visited the dentists for any kind of dental diseases. During this intervention, all the students were required to visit their dentist as part of the annual routine check-up. During follow-up period the use of fluoridated tooth paste was increased and many reports have shown that [31–33] fluoride use is very effective for caries prevention. The relationship between untreated dental carries and consumption of fermentable carbohydrates and sugary diet was also well addressed in our OHE. Several systematic reviews [34, 35] have shown that exposure consumption of fermentable carbohydrates and sugary diet is associated with the prevalence of untreated dental caries. After the OHE, significant improvement was observed for frequency of eating candy/chocolate/sweets (less than 1 time).

Moreover, the active participation of the teachers contributed to the reduction of untreated dental caries among the participants. The present OHE program had arranged training workshops for the teachers in order to reinforcement at follow-up, to provide for exchange of knowledge and experience and to keep motivation high. Some previous OHE program in reducing the dental caries were not reported successful [36, 37] since the teachers received limited instruction on OPHE or they lack motivation. These aspects might have had a positive effect on the good results achieved. Therefore, a school-based OHE program may be exceedingly beneficial for untreated dental caries prevention in Bangladeshi children.

This intervention study provides several importance findings and insights. However, some limitations should be noted. First, the intervention targeted children only at school, not a complete health promotion scenario, as no changes in the home environment were advocated. Second, an inherent bias was most of the outcome variables (practices) were self-reported. Therefore it is possible that, there may be over-reporting of favorable behaviors in the post intervention survey. Third, future studies should determine the cost of up scaling the intervention and the cost saving associated with the intervention promoting preventative dental care. Fourth, although all possible efforts were made to standardize the presentations, it is possible that other environmental factors such as differences in the abilities between RAs in their abilities to disseminate study messages could affect the study outcome.

Fifth, the clinical oral examination in this study only checked untreated dental caries. Some important indicator for adolescent’s oral health such as oral hygiene status and gingival health [38], have not been addressed here. These areas were not addressed because of financial and time constraints. Inclusion of these problems in future research is vital for knowing the effectiveness of OHE program for adolescent’s oral health. Sixth, although we used “school” as a control variable, however, school related characteristics were not available at the time of the analysis. However, an additional analysis was performed by stratifying the data according to the school types, and we found that in all the three schools, OHE intervention had the similar effect. Therefore, it is unlikely that inclusion of such confounders into the model would result in an insignificant link between effectiveness of OHE program on our desired outcomes.

Finally, although OHE program has been successful in many developing [11–14] and developed nations [15, 16] around the world, this approach may produce short-term benefits but fails to achieve sustainable improvements in oral health or to reduce inequalities [39]. Solely focusing on changing the lifestyle of individuals is both ineffective and very costly [40]. Such approach diverts attention away from the causes of the causes, the underlying conditions that cause disease. It is incorrect to assume that lifestyles are freely chosen and can be easily changed by everyone. Health knowledge and awareness are of little values when resources and opportunities to change do not exit. Radically different approach is now needed to reduce oral health inequalities and promote population oral health. Clinical preventive measures and behavioral approaches are not effective at tackling oral health inequalities. Instead coordinated and integrated action is need on the underlying social determinants of health, that is, upstream action to improve living, working and social conditions.

Despite these limitations the results have elicited important information that could serve as a basis for future planning in increasing knowledge, attitude, and practices regarding oral hygiene among adolescent’s school students in Bangladesh through oral health educational promotional strategy. Our study can be generalized to the average Bangladeshi school aged adolescents, firstly, because schools are selected randomly; secondly, in Bangladesh higher secondary education are almost free of cost in both government and semi-government schools and the government of Bangladesh is providing free education up to higher secondary level for the female students. Therefore, adolescents belonging to any socio- economic status can attend the school.

## Conclusions

In summary, this study indicated that the OHE intervention was effective in increasing i) knowledge, ii) attitude, and iii) practices toward oral health; it also significantly reduced the prevalence of untreated dental caries among school aged adolescents from grade 6–8 in a deprived rural area of Bangladesh. Findings suggested that integration of OHE into the general curriculum of training could improve oral-health knowledge, attitude, practices and for prevention of untreated dental caries among adolescent. Long-term value of the improvements associated with the developed intervention approach need to be evaluated in future studies. In conclusion, like many other South Asian nations which share similar socioeconomic profiles and cultural traditions, OHE program was found to be effective in Bangladesh.
